# Promoting Healthy Growth or Feeding Obesity? The Need for Evidence-Based Oversight of Infant Nutritional Supplement Claims

**DOI:** 10.3390/healthcare4040084

**Published:** 2016-11-12

**Authors:** Michelle Lampl, Amanda Mummert, Meriah Schoen

**Affiliations:** 1 Center for the Study of Human Health, Emory University, Atlanta, GA 30322, USA; msschoe@emory.edu; 2 Department of Anthropology, Emory University, Atlanta, GA 30322, USA; amanda.mummert@emory.edu; 3 Department of Nutrition, Georgia State University, Atlanta, GA 30302, USA

**Keywords:** growth patterns, infant feeding, cultural feeding expectations, health advertising, infant formula

## Abstract

The Developmental Origins of Health and Disease (DOHaD) model recognizes growth in infancy and childhood as a fundamental determinant of lifespan health. Evidence of long-term health risks among small neonates who subsequently grow rapidly poses a challenge for interventions aiming to support healthy growth, not merely drive weight gain. Defining healthy growth beyond “getting bigger” is essential as infant and young child feeding industries expand. Liquid-based nutritional supplements, originally formulated for undernourished children, are increasingly marketed for and consumed by children generally. Clarifying the nature of the evidentiary base on which structure/function claims promoting “healthy growth” are constructed is important to curb invalid generalizations. Evidence points to changing social beliefs and cultural practices surrounding supplementary feeding, raising specific concerns about the long-term health consequences of an associated altered feeding culture, including reduced dietary variety and weight gain. Reassessing the evidence for and relevance of dietary supplements’ “promoting healthy growth” claims for otherwise healthy children is both needed in a time of global obesity and an opportunity to refine intervention approaches among small children for whom rapid subsequent growth in early life augments risk for chronic disease. Scientific and health care partnerships are needed to consider current governmental oversight shortfalls in protecting vulnerable populations from overconsumption. This is important because we may be doing more harm than good.

## 1. Introduction

David Barker’s Developmental Origins of Health and Disease model (DOHaD) has led to the recognition of healthy growth as a cornerstone for lifespan health. This framework has been supported by a vast array of epidemiologic evidence linking early life body size to future chronic disease risk and longevity [1]. While body size has long been used as an indicator of health and well-being at both the individual and population levels [2], Barker’s work has brought new attention to the fundamental role of growth in not only reflecting an individual’s health status, but determining it [3]. The importance of aberrant growth patterns during critical periods has been emphasized as a direct link to risks for health sequelae later in life, with higher rates of metabolic disorders [4], for example, among individuals who were born relatively small and grew rapidly in the first years of life [5]. These data bring to the forefront the need to reconsider present modalities of growth promotion among relatively small infants and young children. 

Currently, small children are the target for a large industry of liquid-based nutritional supplements, which claim to ensure the achievement of healthy growth. Although originally formulated to support the enhanced needs of under- and malnourished children, these supplements are now available to the general public and have become appealing “solutions” for parents who are acutely aware of the social [6,7] and clinical implications [8] of small body size. Simply bolstering growth in response to perceptions that “bigger is better” is not without risk, and the long-term health ramifications of feeding supplements are becoming increasingly clear [9]. Concerns about increasing weight without increasing skeletal size have been raised amongst human growth specialists since 1959 [10]. A half-century later, a substantial evidence base from animals and humans alike has demonstrated that supplementary feeding efforts may grow plump babies rather than building larger skeletons clad with muscle [9,11]. In a world of increasing obesity-related chronic disease [12], this is an undesirable consequence. 

Now that supplements have entered the marketplace for children-at-large and the number of children consuming them during critical periods of growth continues to increase, it is essential to consider the evidentiary base supporting the health claim properties of liquid-based nutritional supplements. Furthermore, it is of interest to assess how these statements are modifying social beliefs and cultural practices surrounding feeding in infancy and beyond. An overview of the scientific evidence behind the statement “proven to help kids grow” reveals an opportunity for improvements in oversight and questions the relevance of such statements for otherwise healthy children. This is important because we may be doing more harm than good.

## 2. Promoting Healthy Growth: The Use and Claims of Liquid-Based Nutritional Supplements

Liquid-based nutritional supplements exist for all age groups, with a multi-billion dollar market specifically developed for infants and young children globally [13]. Often referred to as “follow-up formula” (FUF), “growing-up milk” (GUM) or “toddler milks”, these liquid-based “nutritional supplements” have great similarity to infant formulas, but are intended to function as meal complements rather than meal replacements [14]. A number of fortified beverages are predominantly targeted for the transition and weaning periods, with FUFs developed for children from six months of age as part of the weaning diet and GUMs, or toddler milks, intended for children from one to three years of age. Prepared from the milk of cows, sheep, goats, or other constituents of plant origin, both FUFs and GUMs may have modified protein content and composition, in addition to a range of added nutrients (e.g., fatty acids, micronutrients, probiotics, prebiotics, or synbiotics) [14]. These products are currently sold to a wide customer base in what was estimated in early 2016 to be the “fastest-growing packaged food market” globally [15].

Liquid nutritional supplement products among infants were, however, originally developed as a supplemental source of energy, proteins and/or specific nutrients for under- or mal-nourished children [16] and those diagnosed with “failure to thrive” (FTT), a non-specific diagnosis of growth failure stemming from a range of biological, psychosocial, and environmental factors [17]. Severe malnutrition is accompanied by pathophysiologic and adaptive changes that raise unique feeding challenges for nutritional rehabilitation. These include fluid, electrolyte, mineral and vitamin imbalances, and issues of gastrointestinal absorption, immune competence, and thermal regulation that alter nutritional needs during recovery [18,19,20,21]. The special needs for recovery from malnutrition are further accompanied by a pattern of increased weight prior to, but not necessarily followed by, increased length growth [22].

Despite this history, a number of such supplements have expanded beyond the clinical realm and are available “over-the-counter” for consumption by infants and children regardless of their actual nutritional status, size and health state. They are part of a genre of products identified as nutritional support for “healthy” growth overall. Displayed in both the formula and health food aisles in supermarkets, FUFs and GUMs are packaged, branded, and labeled similarly to infant formula and general foods, making it immensely difficult for consumers to distinguish between the products [23]. Perhaps more importantly, there is no explicit rationale as to why a consumer would want or need to distinguish between them. Fostering health through promoting growth is a sensible goal that is within the vision of what most parents want for their children. What parent would not consider a product that promises to “help kids grow” [24]? There is, however, a problem with the appeal. What does it mean to “grow”? If growing is gaining weight, many children at this time do not need “help” and “growing bigger” is not necessarily healthy. Thus, identifying who will benefit from extra calories and/or other nutrients is key to implementing practices that promote health by stimulating growth, not simply stimulating weight gain which is often predominantly added fat.

## 3. The Importance of Initial Conditions: Smallness

The original target for a number of liquid supplementation products was clinically small individuals for whom smallness was associated with health risks. When suffering from poor nutrition, illness, physiological or anatomical perturbations, and/or behavioral issues, some children stop growing. This is not merely a matter of overall size but extends to organ structure and function, with implications for long-term health [25]. Interventions aiming to turn the tide and stimulate growth, and thereby development, can be seen as health promoting due to their associated decreased risks of morbidity and mortality. 

The challenges in any intervention aiming to promote bigger size among smaller children include, first and foremost, the definition of “small”. While arguments ensue as to what defines individual smallness, the diagnostic reference frame is presently either an age-based comparison among peers of common background in order to control for environmental influences [26], a national reference point [27], or a global size-for-age standard assuming an optimal environment [28]. These frameworks recognize the importance of a broad range of environmental modifiers of growth biology. The clinical definition of “small” is based on a statistical criterion. Commonly used cut-offs in the United States have included someone who is either smaller than 95% of others in a similar age group, thus below the 5th percentile for age, or less than two standard deviation units below the median [29]; while globally, the definition is applied to someone smaller than 97% of others in a similar age group, or below the 3rd percentile of the WHO growth standards [28]. 

All “small” infants and children are not, however, equal. Individuals under the 3rd or 5th-percentile for age, for example, do not share a common set of conditions. Some children may be “normal small” because everyone in the family has always been small (genetic programs at work), while a wide range of causal pathways that may pathologically inhibit growth (including but not limited to nutrition, disease, or eating behaviors) can result in an individual being smaller than would be the case in the absence of these conditions.

*Size is not growth*. Growth is change through time; size at any time point summarizes changes accrued previously but offers no insight into the incremental process itself. Only repeated measurements across time can document growth [30]. This is important as children do not grow at the same rates according to the same time frame [31]. The decision regarding who may benefit from interventions for smallness is not necessarily simple. This can be better clarified by determining how an individual is small—in what dimension they are small and due to what process.

*Measurement specificity matters*. Some children weigh less than peers of similar age, others are “short”, and some are “thin”, or of relatively low weight for length or height. These measurements are not, however, interchangeable markers [32]. Instead, these parameters represent distinct physiological pathways involving differences in both metabolism and skeletal accrual. The most commonly used parameter, weight, is not actually a growth proxy at all [32]. Weight gain or loss represents the balance between energetic intake and utilization, and the consumption of calories above that which can be used accrues as body fat. This phenomenon occurs among adults who have completed their physical growth, and may increase plumpness, or weight-for-height. This is often captured by body mass index (BMI), a type of morphological expansion not equivalent to increase in overall skeletal size, the process that takes individuals from infant dimensions to adult height. These details are important. As a principle of growth during development is increase in size across time, it is important to specify what is increasing. While getting bigger in terms of weight gain may be instrumental in reducing risk of mortality among infants in the neonatal intensive care unit or those recovering from severe malnutrition [22], it may augment health risks among individuals in alternative conditions [33].

## 4. Growth and Health

Equating getting bigger with health is not a universal law. It is a specific case. Claiming that a nutritional supplement promotes “healthy growth” is a specific statement that requires a conditional framework involving the dimensions measured, the time frame of change, and the initial conditions. For example, under conditions that inhibit growth and result in small size, introducing an intervention that releases the inhibition and permits the system to grow is arguably beneficial. These are the circumstances under which a number of nutritional supplements were introduced. According to the citations accompanying product descriptions, the original goal of a number of products was to assist children whose growth was restricted by insufficient or poor diet, disease, or altered eating behaviors. In these contexts, small size was a macro-level indicator of risk for perturbed development that may permanently alter structure and/or function compatible with compromised health [25]. In the absence of intervention, morbidity and mortality risks may increase. In this scenario, an outcome of “bigger” was seen as a potential risk reduction, or promotion of health. A closer look at the studies supporting one such product provides perspective on the importance of these specifics.

## 5. The Scientific Rationale for the Claims: The Nature of the Evidence

A comparison of five different studies referenced on a commercially-sponsored website [34] provides a case study [35,36,37,38,39]. Collectively, these studies provided a concentrated liquid supplement (1 kcal/mL; 12% protein, 44% carbohydrate, 44% fat) and are cited to support the statement “clinically proven nutrition to help kids grow” [34]. Two words merit close consideration: “proven” and “kids”. Taking a look first at the samples representing “kids”, it is important to note that the statement fails to specify just what group of “kids” were studied, e.g., their initial conditions and growth status prior to intervention. Those groups of “kids” included: 

1. *Clinically documented under- and malnourished children.*

After 14 days, 30 of 40 severely malnourished Pakistani children aged 1 to 5 years gained weight and accrued fat [35]. 

After 8 to 35 days of feeding, 13 children suffering from kwashiorkor gained weight, and after 2.4 months of feeding an unspecified amount of the target product, nine 6–40-month-old children recovering from protein-energy malnutrition weighed more and were longer [38] .

2. *Chronically disabled children who require specialized nutritional support and total enteral support by oral or nasogastric feeding.*

Among 14 children, increased height, weight, and skinfold thickness was observed after 12 weeks of feeding the product, which provided ≥90% of daily nutrition in a nonrandomized study that lacked a control group [39].

3. *Relatively small individuals defined by low weight-for-age or weight-for-length samples.*

After four months’ intake, the average weight-for-height changed an average of 10 percentiles among 626 children who were 1 to 3 SDs below the median weight-for-height at study onset [37].

4. *Picky eaters.*

Ninety-two “picky eaters” aged 3 to 5 years with “evidence of growth faltering” were randomized to receive either nutrition counseling alone, or nutrition counseling plus the nutritional supplement for 90 days [36]. The latter group gained more weight and height than the unsupplemented, counseled group.

## 6. Kids: Sample Specifics

None of these five samples focus on generic “kids”. Instead, all samples embody the fact that under- or malnutrition (due to multiple causal pathways) inhibits weight gain and impairs linear growth, resulting in relatively smaller body size. Feeding releases the inhibition and supports tissue accrual, allowing such children to gain weight, and sometimes, to the extent they were inhibited from overall growth, subsequently increase other body parameters as intake increases [22]. As an interval of “catch-up growth” is normally found among such samples [22], all study designs represent sampling bias, which precludes identifying whether a *specific* feeding protocol is associated with subsequent increased body size. 

## 7. Proof: Intervention Causality

It is worth considering what, exactly, was “proven” in these studies. As observational studies cannot prove causality, and the two studies described as randomized control trials (RCTs) did not actually randomize for the product itself, these studies are all merely observational. The first three studies previously outlined describe observations from fewer than 60 ill children with no control groups [35,38,39]. The latter two studies, designed as RCTs, do not actually control for the supplement itself. Instead, Fisberg et al. [37] offered the nutritional product with and without an added fortified synbiotic (which had no significant effect on the incidence of illness, number of sick days, or number of sick episodes); Alarcon et al. [36] offered the nutritional product in addition to nutritional counseling, as a comparison to the control group that was provided counseling only. 

Beyond inconsistencies in study design, there was no singularity of consumption. “Doses” ranged from a 14-day intervention among children who consumed the product “according to demand and tolerance” while also receiving breast milk [35] to a provision of at least 90% of all daily nutrition needs for 12 weeks [39]. One might suggest that this collection of studies fails to provide robust proof of principle on any point, suffering from sub-optimum design due to biased samples, absent or inadequate controls, and questionable criteria for outcome assessment. Thus, causality cannot be identified even in these clinically growth-impaired children, much less be extended to normal children.

## 8. Outcome: The Evidence and the Claims

Across time, infants and children normally get bigger, albeit by very different paths and rates [40]. Simply becoming “bigger” across time provides no evidence of product-specific efficacy. These studies show that the interpretation of meaningful outcomes after interventions aiming to promote growth is an area in significant need of review, standardization, and oversight. For example, there is no biological rationale either for the concept that the 50th percentile of the NCHS reference should be a goal for an individual’s height or weight [38], or for attaching significance to a change of 10 percentile points at the group level [37]. The latter is neither surprising nor compatible with an interpretation of “catch-up” growth [41]. The misappropriation of auxological terminology here (catch-up growth) leads to claims that are not evidence-based.

In summary, a review of the studies cited to support an evidentiary base for “clinically proven nutrition to help kids grow” questions whether anything was actually “proven”. The samples were certainly not “kids” generically, but those whose initial conditions were associated with known unique physiologies. The transmutation of the limited study findings to support generalized marketing to healthy normal children is a leap based on significant omissions. While this may not be a legal breach, perhaps it is time to question whether this makes it an acceptable practice and if further oversight might be beneficial. This is important because a plethora of liquid-based nutritional supplements are presently being marketed to otherwise healthy children of “normal” size.

There have been a number of calls to stop misleading health claims among advertisements of infant and children’s food products [42]. The semantic paraphrasing in many marketing pitches is more than a packaging issue and more than “puffing”. The requirement that all labeling be truthful and not misleading is worthy of review in more than a few cases. The operative word here is misleading. This type of practice is well known in the industry and other products have not escaped controversy [43]. An increase in oversight going forward will involve assessing the accuracy of a number of product claims by clarifying the strength of the data upon which they are based and the assumptions upon which the interpretations rely. This includes identifying what the term “growth” refers to (e.g., height, weight, weight-for-height) and assessing both how the concept of “normal” growth is employed and the validity of study designs (sample size, intervention duration, and data analytics). There is a scientific knowledge base against which evidentiary claims can be assessed that is not being thoroughly considered. Given the human stakes, this may require more than reliance on reviewers and editors, and calls for more formal oversight.

## 9. Assessing “Healthy” Growth Structure Function Claims

### 9.1. The Use and Misuse of Growth Charts

Identifying the parameters of “normal growth” scientifically has been based primarily on descriptive criteria, emanating from the work of auxologists, scientists who study the size of children—measuring them, calculating descriptive statistics by age, and devising both tables and graphical images of size-for-age so that the relative size among peers of similar background and sex may be quickly appreciated [44,45]. These reference data sources include the growth charts found among the working tools of clinicians, community health workers, and others involved in monitoring child health and well-being. The utility of growth charts are often misunderstood. They are not actually intended to function as sole diagnostic criteria, but as references [27] or standards [28] for identifying and noninvasively monitoring potential health or nutrition-related problems, summarized at the whole body level as growth perturbations. On these charts, “normal” size for age has a wide range and is generally interpreted as sizes falling between the 5th or 10th and 90th or 95th percentiles (or 3rd and 97th, by way of the WHO standards), with the caveat that, by definition, some people are among the statistically relatively small (<the 3rd, 5th, or 10th percentiles) and/or large (>the 90th, 95th, or 97th percentiles). 

While graphical presentations of these statistics are often assumed to represent “tracks” for children’s changing size across time, this is not actually what they show. In fact, they are not designed to provide this information. Growth charts are not an amalgamation of the paths actually taken by individuals. Instead, they summarize data on children’s sizes at targeted ages. By analogy to a connect-the-dots schema, the growth curve lines connect empirically derived descriptive statistics of size for age, joining similar percentiles (e.g., 3rd, 10th, 50th, 75th, or 90th) across ages. This results in easily readable interpolations of the relative size of children at each age, but does not record the actual paths by which individuals grow across time [30,31]. While some individuals may grow at rates that keep them close to these lines, many do not. This is because children grow neither continuously [46] nor at similar velocities. Individuals grow more frequently at some ages than others and some people grow more frequently and/or with greater amplitude than others. It is entirely normal for individual children to grow at variable rates [40], which results in them being relatively shorter or taller, heavier or lighter than their peers, irregularly across ages. A relatively short four-year-old may become a relatively tall five-year-old, for example, and this biological growth trajectory may take him from a size at the 35th percentile among four-year-olds to the 60th percentile among five-year-olds a year later. This would result in a pictorial “percentile crossing” pattern when plotted on a growth chart. 

### 9.2. The Construction and Perpetuation of “Healthy” Growth Structure/Function Claims

These fundamental biological facts are often misunderstood and/or mis-stated. A consequence of this includes erroneous assumptions regarding “healthy growth”, which in turn form a basis for invalid claims of efficacy. An example of this is the claimed “success” of a novel approach to promoting infant growth by “enhancing the infant growth rate over the expected rate” for weight, height, and/or head circumference in the first six months of life [47,48]. In this example, the “expected rate” was embodied by the Z-score at birth. The null hypothesis was that “the natural growth of each infant is expected to remain on the same percentile; namely, the Z-score is expected to be stable during growth” [48] and “the effect of the supplemented feed was evaluated as the difference between the Z score at 6 months and that at birth” [48]. The problem here is that this is not biologically accurate and, thus, the premise on which the study design was based is incorrect. 

As clearly documented in 1976, infants do not remain at the same size percentiles from birth to six months of age [49]. If they did, prenatal growth rates would determine size for life and all children would have to grow at the same rates across time to maintain their sizes relative to one another (percentile rankings of size-for-age). Individual growth biology does not unfold like this. A common example is the observation that newborn size is often constrained by maternal factors prenatally, and a relatively small neonate can undergo rapid postnatal growth when these constraints are alleviated. A rapid size increase, compared to peers of similar age, translates to a relatively larger size and a correspondingly higher percentile of size-for-age. The opposite can also occur, with relatively large neonates “slowing down” their growth rates after birth. As reported by the United States CDC in 2004, nearly 60% of individuals under the 25th percentile at birth (the study sample characteristics) change Z-scores over the first six months, crossing percentile lines in weight and length, and more than 70% cross percentile lines in weight-for-height [50]. Thus, the null hypothesis proposed in this product test case was incorrect, both at the time of the study and its report. Normal growth is not defined by adherence to a constant Z-score across age. As any discrepancy between a Z-score at birth and six months of age exemplifies the false criteria fallacy, any interpretation is meaningless. This is quite apart from the fact that the Z-score reference employed, the CDC data base [51], does not actually include data from infants within the age range of the study sample, and that the validity of the *mean* result across 10 study subjects is a questionable outcome, as some individuals were characterized by increasing and others by decreasing Z-scores across time [48].

Misunderstanding of growth curves and how they should be used in individuals is all too common. For several dietary product entities in infant and child nutritional supplement portfolios, beneficial growth outcomes are inappropriately assessed and claimed. Statements of this nature, which describe how a dietary supplement “affect(s) the normal structure or function of the human body”, is a structure/function claim [52]. As a number of liquid supplement products are positioned as helpful to children’s “growth”, it is time to consider the nature of the evidence upon which efficacy is said to be substantiated. Increased scientific scrutiny and oversight of such claims are needed for the well-being of our youngest citizens.

### 9.3. Providing Informational Health Material 

Structure/function claim inconsistencies are not, however, the only area in need of review. Marketing-based ancillary materials may also be factually incorrect. It is worth considering what role product marketing *should* be playing in the transmission of health-based information to the public. Auxiliary information, both online and in printed materials, is frequently offered to provide guidance, particularly among direct marketing enterprises. An example of this involves resources made available to address questions that may influence the consumer’s choice of a supplement. Most direct marketing sites provide clear statements directing consumers to consult with their pediatrician regarding whether or not an infant, toddler, or child nutritional supplement is an appropriate choice. This removes any overt culpability on the part of the product providers for consumers choosing to purchase the product. These online materials may also aim to educate consumers or support them in their interactions with their physicians by offering a “translation” of what the pediatrician might say during a consultation, for example. All of this would appear to be well aligned with efforts to properly inform the public in decision-making. It may also be an effort to empower consumers by offering a sense of reassurance, trust, and support in product selection.

One such example includes resources found on an industry-sponsored website [53] in a “get the facts” section aiming to provide consumers with information on “healthy” child growth. Here, text explains that pediatricians assess children’s growth by comparison with “the expected healthy growth pattern exhibited by millions of children considered healthy and growing as they should” in which “the growth chart displays curved lines that represent the expected pattern of gains in weight and height (or length) for children growing as expected”. Problematically, neither of these statements is accurate. For the growth chart pictured on the web page, only size at birth comes from a large sample: the United States Vital Statistics (1968–1980; 1985–1994) provides data from more than 40 million boys and girls who were born over 1500 grams (healthy or not). However, the growth chart curve itself is based on far fewer individuals, including data on zero to 30 individuals at some early ages [48]. Zero subjects means no actual data, hence no information on the size of actual children. The curvilinear lines are, once again, merely mathematical imputations, connecting dots where data do exist by interpolation. Not designed to show “expected patterns” of weight and height gains, the product “information” site misstates even its own reference [54] on how to use the growth charts. 

One might ask, why do any of these details matter? First, providing factually incorrect information under a “get the facts” tab is fundamentally problematic. Perhaps more importantly, this is a missed opportunity to educate the public. Suggesting that healthy growth patterns are known from millions of children provides a false sense of knowledge. Quite the contrary, identifying who can actually benefit from consumption of nutritional supplements is not at all clear. Offering information of this type fuels the perception that the seller shares in a sphere of expertise that can be relied upon. These types of trust-engendering statements may not be needed to sell a particular product, but they are important to build trust in the brand. While there may be no reason for a marketing team to be experts in human growth, it appears to be time for more validity oversight. Accountability regarding scientific realities is needed through policy. 

## 10. Marketing Health

### The Emergence of Infant Health Foods

The marketing of infant foods is a unique niche. Successful parent-directed marketing of infant and young child food products is embodied by the iconic Gerber baby image trademarked in 1931, a brand that remained synonymous with trustworthiness and quality as demonstrated by higher reported consumer loyalty than any other commercial brand in the United States in 2002 [55]. Sold to Nestlé in 2007, the Gerber name was retained for its product line and was the leading baby food and snack brand in the USA in 2016 [56]. Gerber products aim to introduce infants to food and “encourage the development of healthy eating habits” [57]. This long-standing brand offers a developmentally grounded product line, aligning biological indicators of motor development from birth through supported and independent sitting, crawling, walking, and preschool ages, with foods that change in texture, taste, and accessibility to match infants’ emerging skills. 

In contrast to this maturationally based support of food introduction, the emergence of infant liquid supplements targeting the time of weaning and complementary food acquisition aims to continue the developmentally earlier reliance on liquids. Rather than being left behind a post-formula maturational rubicon, formula companies have used cross-promotion and “brand-stretching” to expand their product portfolios with “toddler milks”. Employing an age-based sequence of product progression, new subcategories of formulas have emerged, offering liquid-based “supplements” to bridge the weaning transition and track the young infant into childhood, with some of its products targeted for ages one to 13 years [34]. 

Following the rise in the adult liquid nutritional supplement markets, perhaps this infant product development and expansion is not surprising. Consumption of enhanced waters and health-specific drinks touting all manner of benefits, offered in easy-to-consume packaging for on-the-go lifestyles, has been embraced by the same consumer population that is targeted by the infant supplement products. In a climate of health-oriented nutritional awareness in general, toddler milks are a new “health product”, with claims of developmentally specific, nutritionally important ingredients. This is in spite of the fact that the World Health Organization has deemed such products unnecessary and potentially harmful to children’s long-term health. Touting health benefits in what is perceived as a “science-based” milk that is easy to use among busy mothers and active children has extended the reach of these products into a growing consumer market globally, particularly in Asia [58].

Many of the new products’ efforts to create a health brand are tied to product claims for specific measurable growth outcomes, and these have been shown to be important influences on parental purchasing and product use. For example, a study among a group of Australian parents, asked to recall advertisements they had seen for both infant formula and toddler milks, documented that more than half of the parents surveyed recalled the marketing materials’ descriptions of the fortified milk product as “ensuring proper growth and development” [59]. The successful entrance of these products into the cultural mindset of parents who may adopt such options as part of a healthy lifestyle is worth further consideration. 

Much of this acceptance can be attributed to the genre of marketing associated with structure/function claims, leveraging product appeal for consumer potential. The global baby food market was estimated at $55 billion in 2015, up 8% since 2010 [60]. Two multinationals account for 80% of U.S. sales, with pediatric nutrition product net sales ranging between two and four billion dollars in 2015 [60,61,62]. The escalating use of follow-up formulas globally includes provision to infants younger than national legislation and international recommendations, without best practices for basic hygiene, raising safety concerns for infants under two months of age [63]. This is taking place with a relative lack of marketing regulation. While the World Health Organization released recommendations on the marketing and promotion of foods and beverages specifically for children in 2010 [64], and followed this with recommendations for infants and preschool children in 2015 [65], there remains no specific oversight of structure/function claims for infants and children at this time, and little accountability. While no doubt they should, the average consumers do not investigate the details of the supporting evidence for structure/function claims, which are often identified by asterisks in marketing materials. 

## 11. Sociocultural Implications of Liquid-Based Nutritional Supplements

Despite the questionable evidentiary base supporting the structure/function claims of liquid-based nutritional supplements as they are being used at this time, these products are largely changing the landscape of infant and early child feeding. Beyond convincing parents that these supplements are beneficial for their child, the products and their positioning have instilled the belief that they are *necessary*, providing superior nutrition compared to breastmilk [66]. The Infant Feeding Survey (IFS) from the United Kingdom, which reports a plethora of reasons why parents have chosen to introduce follow-up formula into their child’s diet at 4–6 months of age, found the primary response was that follow-up formula provides more nutrients and is better for the baby than both infant formula and cow’s milk [67]. In addition to the perceived nutritional importance of these products, parents in Luxembourg also describe behavioral benefits, reporting that follow-up formula increases satiety and aids their infant in sleeping through the night [63]. Further explanations for the use of these types of fortified products comes from Chinese mothers in Singapore, who believe that certain follow-up formulas can enhance their infant’s Intelligence Quotient (IQ) [68]. As these reports evidence, many parents globally believe that utilizing toddler milk rather than exploring other complementary foods is a good solution, in fact a healthier option.

Furthermore, the consumption of these products is an easy solution to infant and child feeding, alleviating parental worries of daily nutritional adequacy for their children. This is particularly the case when a child is “picky” towards food, a behavior with reported prevalence rates of 19% among infants 4–6 months of age and 50% among infants 19–24 months of age, as judged by a nationally representative random sample of more than 3000 parents in the USA [69]. Although it can take up to 20 times of trying a food for a child to decide if they like it, most mothers offer a new food fewer than five times and then switch to a more “convenient” feeding option [70]. With this choice, parents reduce food variety in their child’s diet and delay the important transition to family foods. This is associated with increasing potential for food neophobia [71]. Constant intake of the same milk supplement can stunt the maturation of a child’s flavor preferences and, ultimately, the nutritional variety that they are receiving from their diet [71]. As food habits developed in infancy have been demonstrated to track into adulthood [72], and may have long-term metabolic consequences [70], the growing “commerciogenic malnutrition” from formula products raises new health concerns [73,74]. These include the predilection for high energy-density snacks, limited consumption of fruits and vegetables, and the development of obesity later in life [70]. 

## 12. Feeding Obesity

### 12.1. Nutritional Content and Consumption Patterns

Amidst the rapidly expanding global markets for these child dietary products, it is important to consider the consequences of consumption. One perspective comes from a report issued by an international expert panel convened in 2014 to address the lack of international oversight regarding FUF and GUM nutritional composition [75]. The consensus opinion began by calling for a stop to the use of the term “growing up formulas” “in the name of the products or the product category because no specific effect on growth has been shown” [75]. Instead, the term follow-up formula for young children, FUF-YC, was proposed. In agreement with the conclusions of the European Food Safety Association (EFSA) [76], this expert group concluded that FUF-YC can contribute to “improving the supply of critical nutrients, overall nutritional status and hence help to support child health” when “comprised of an appropriate composition, under appropriate conditions of use among one to three year olds”. The committee further specified that desirable levels of nutrients in FUF-YCs include a range of 45 to 70 kcal per 100 mL as an appropriate energy density to avoid significant risk of excessive weight gain and development of obesity, with daily levels per 100 kcal of 1.6–2.7 g protein, 4.4–6.0 g fat, 9–14 g carbohydrate, and a minimum of 200 mg calcium [75]. Subsequently, the joint FAO/WHO food standards programme Codex committee on nutrition and foods for special dietary uses took up the same topic and proposed that follow-up formulas for young children (12-36 months) shall contain not less than 60 kcal and not more than 70 kcal of energy per 100 ml [77], leaving for further discussion whether the lower limit should be 45 kcal/100ml for children more than 24 months of age. 

These committees’ conclusions provoke consideration of potential outcomes from products already on the market, differing in formulary and suggested consumption patterns from these recommendations. One example involves a FUF-YC product specifically marketed as “Nutrition to help kids grow” [78] and presently advertising its significance as a “2 per day” snack, helping “kids ages 3–4 grow out of at-risk weight-for-height percentiles” as it “helps kids gain weight in just 8 weeks” [79]. The corporate-sponsored study cited as the evidentiary base is an intervention with no control arm, involving 199 Filipino children with baseline mean age of 41 months (range, 3 months to 48 months), weight-for-height of 14.8 and 17th percentiles (female, male), and daily intake of 1379 kcal [80]. The authors define individuals between the 2.3 and <25th percentile in weight-for-height as at-risk of wasting. There are three notable points regarding the sample and its attributions: three-month-olds are outside the intended age range for the product without medical oversight [81], by no scientific definition is “at risk of wasting” accurately applied to individuals between the 2.3 and <25th weight-for-height percentiles, and the daily caloric intake is significantly above other estimates of daily energy intakes among either Filipino three-year-olds (941 kcal) [75] or the recommendations for three- to four-year-olds in the United States (1000 to 1200 kcal) [82]. The study protocol involved offering two daily servings (240 kcal each) of a 1 kcal/mL supplement, or 480 additional daily kcal, for 48 weeks. The children’s actual caloric consumptions during the study were estimated from 24-h recalls at the assessment points as an additional 270 kcal per day at four weeks, increasing by a further 136 kcal at eight weeks (for a mean 1786 kcal per day), followed by a plateau thereafter with attenuated increases to a total of 500 kcal above baseline at 48 weeks (an average of 1956 kcal per day). The intervention was associated with a total weight gain of 0.5 kg after four weeks, 0.8 kg at eight weeks, and 0.2 to 0.3 kg per eight weeks thereafter, for a total accrual of 2.2 kg at 48 weeks. The product website further touts that “2 per day” is associated with “improved height and weight-for-height” after 24 weeks. This “improvement” was a mean increase of 3.5 cm in height (a 0.12 Z-score change) by week 24 (accrued as 0.5, 0.7, 1.2, and 1.1 cm sequential increments at 4, 8, 16, and 24 weeks) and a weight-for-height Z-score (WHZ) change of 0.46 by week eight. The authors conclude that these results show a catch-up growth phase in weight followed by a maintenance growth phase of height gain, a successful “preventive approach of targeting children at risk of undernutrition” with “significant benefits on growth” and “low risk of excessive weight gain” as the WHZ change was below 0.67 [80].

This report raises a series of scientific points: (1) *Children normally increase in size through time*. As the sample mean height increments are not unusual for three- to four-year-olds between the 10th and 15th percentiles of height-for-age [28] (the baseline sample characteristic) and there is no control group, no product-based “growth” effects at any age can be claimed. (2) *The consumption of excess calories leads to weight gain and weight gain is not growth*. Contrary to the conclusions of the authors, the data cannot describe catch-up growth [40,83] and do not describe saltation and stasis growth [46]. Instead, the weight gain pattern of acute increase followed by plateau is expected as a consequence of an abrupt step up in caloric intake and subsequent adjustment by, and response to, increased weight [84]. The reported magnitude of weight gain is compatible with the extra energy intake as empirically predicted by a mathematical model of weight gain in children [85]. With a supplement providing 100 kcal/mL, which is substantially greater than the 60-70 kcal/ml upper limit recommended by the expert panels to protect against inappropriate weight gain and obesity [75], the authors’ dismissal of potential risks attendant to weight gain effects deserves further consideration. 

### 12.2. Consequences for Weight Gain

The categorical criterion employed to rule-out concerns of inappropriate weight gain, 0.67 increased WHZ scores [80], is the distance between two major WHO growth standard percentiles [86]. While frequently used as a predictor of subsequent risk for overweight and obesity at the group level [87], 45% to 50% of three- to four-year-olds do not cross one or more weight-for-height percentile line(s) [50]. The use of this simple dichotomous variable as a definitive diagnostic to accurately identify processes that will unfold at the individual level over time at this age is empirically poor. Errors commonly accompany the use of categorical summaries of continuous data as graded responses are lost [88]; smaller alterations may well be initiating longer term trends. A study of 578 children found that those with WHZ score changes less than 0.67 between 12 and 23 months of age had a higher prevalence of subsequent obesity by age four to five compared to their peers who experienced changes in WHZ scores of 0.67–1.0 [89]. This simple percentile-crossing criterion is insufficient to safeguard children from potential sequelae due to weight gain from high calorie intake and should be rejected as proof of safe usage. 

A number of lines of evidence point to associations between graded increases in weight, weight-for-height, and BMI during early childhood and biomarkers of health sequelae. A review of 25 studies identified increasing z-scores in weight gain between one and four years of age to be significantly associated with increasing BMI, adiposity, and percent body fat in mid-childhood [90]. Similarly, increasing BMI z-scores between age two and three are found to be associated with increasing biomarkers of mid-childhood cardio-metabolic health, including increased percent body fat, independently of birth size effects [91]. The age from three to five years is emerging as a critical period for childhood growth trajectories that precede obesity and later chronic disease, including increased blood pressure and diabetes [92,93,94]. These data raise serious questions regarding the health implications of high-calorie liquid dietary supplement consumption among young children. 

While there are no doubt acute events that compromise nutritional intake and lead to severe weight loss from which children may benefit from a rapid turn-around in weight gain, these are likely to necessitate pediatric oversight and are not appropriately resolved by direct marketing products. For the average over-the-counter consumer, it is unclear why it is desirable for children to gain weight in “just 8 weeks” [78]. The website clarifies that among the evidence base for the product claims, “Over half of the children grew out of at-risk weight-for-height percentiles”, adding in a footnote that this referred to children “in the 5th–25th weight-for-height percentiles” [78]. Herein lies a problem: 20% of healthy children are normally between the 5th and 25th percentiles in size for age; these are not automatically considered “at-risk” percentiles by any international convention. In a world of rising childhood obesity, perpetrating a need for weight gain among healthy children who are below the 25th percentile in weight-for-height is potentially harmful. This marketing strategy plays on well-documented perception errors of mothers regarding their children’s size [95] and is likely to exacerbate rather than alleviate the global child overweight and obesity trend. 

### 12.3. Feeding the Obesity Epidemic

Thus, in addition to individual level health consequences, public health issues are also at stake. Using retrospective epidemiological data, a mathematical model of childhood energy balance and bodyweight dynamics that accounts for healthy growth and development [96] has proposed that at the population level, the excess weight of U.S. children in 2003–2006 compared to similar children in 1976–1980 was associated with a mean increase in energy intake of roughly 200 kcal per day per child. At an individual level, the same approach estimates that a child at the 50th percentile for body weight at the age of five, consuming an extra 400 kcal per day over the next five years, would become 10 kg overweight by comparison with a peer who did not. Given the suggested suitability of some of these products for consumption between ages two and 13, it is yet to be seen what the consequences may be for child weight profiles globally in the coming years. 

## 13. Oversight Challenges

The absence/lenience of governmental regulations on liquid-based nutritional supplements is among the driving factors behind the altered perception and practice of infant and child feeding [66,70]. The most significant piece of legislation that created boundaries on the advertising and use of these products was the “International Code of Marketing of Breast-Milk Substitutes” [97]. Adopted by the World Health Assembly in 1981, this code attempted to define appropriate avenues of marketing and distribution for formula products by prohibiting their advertisement when represented as total or partial replacements for breast milk. Moreover, the International Code provided additional provisions to protect “vulnerable populations” or groups that are more likely to succumb to advertising campaigns due to increased exposure in the marketplace, less education, or less experience [98]. Children and parents may be considered vulnerable populations in the arena of health marketing. Newborns, infants, and young children have little input regarding their consumption patterns and are susceptible to marketing following what their caretakers hear is “best” for their child [66,99]. These specific provisions included the prohibition of breastmilk substitute promotion directly to mothers, restrictions on contact between company employees and mothers, and constraints on interactions between formula companies and health professionals [97]. In order to carry out this legislation, the World Health Organization declared that formula companies must first demonstrate that their advertising will have no deleterious effects on breastfeeding before they can begin any widespread advertising campaigns [97]. 

Despite the positive intentions of the international code, the United States has not taken steps to implement it. This is evident in the fact that there remain no legal restrictions on the advertisement of infant formula add-on products [100,101]. In fact, follow-on formula began to be promoted more aggressively following the introduction of the International Code, as demonstrated by the publication of the first standard for follow-on formula composition, also in 1981 [102]. It has been postulated that follow-on formula may have been created to evade the provisions of the International Code because it is specifically marketed as a complementary food for infants at the weaning age, rather than as a breastmilk replacement [100]. As practice often does not align with this marketing, there is immense controversy as to whether follow-on formulas and other liquid-based nutritional supplements fall within the scope of the International Code [74]. 

Beyond a lack of advertising restrictions, infant and child dietary supplements are also not subject to the general food safety regulations that control commercial infant formulas. This is due to the Dietary Supplement Health and Education Act (DSHEA) of 1994, which determined that dietary supplements do not require FDA approval [103] and that the FDA could not demand scientific proof if no claims were made regarding “treatment”, “prevention”, or “cure” [52]. This framework was further enhanced by the Food and Drug Modernization Act (FADMA) in 1997, which permitted structure/function claims to be included in marketing, with terms such as “support”, “promote”, and “maintain” allowable in statements linking a product’s role in maintaining normal function, with the caveat that consumers must have a reasonable expectation of no harm [104]. Despite their rule that disclaimers must be present describing the state of the evidence used to substantiate the statement, these laws placed the FDA in a reactionary rather than gate-keeper role regarding the truthfulness of claims for nutritional supplements. Leaving matters in the hands of industry to monitor its own adherence is unproductive as they are both well aware of the regulations and cognizant of the potential enforcement as a threat to business expansion [62].

Additional problematic aspects of oversight involve the inclusion of “functional” ingredients in toddler milks with the intent of targeting specific biological effects. Examples of such ingredients include long-chain polyunsaturated acids such as docosohexanoic acid (DHA) and arachidonic acid (ARA), which have been postulated to be on the causal pathway of enhanced intellectual development among breastfed versus formula-fed infants [105,106]. It remains unclear whether many of these ingredients fall under the category “generally recognized as safe” (GRAS) due to the fact that efficacy is not a condition of GRAS affirmation [107]. 

Finally, while the WHO has long maintained that follow-up formulas are not necessary for proper infant or child nutrition [108], the FDA’s release of guidance on “Distinguishing Liquid Dietary Supplements from Beverages” indicates that there is a growing concern over the representation of liquid-based nutritional supplements [109]. As infants’ food intake is largely liquid across the first year of life, it may be time to abandon the distinction between marketing for infant “foods” and “supplements” and to specifically require pre-market approval for items targeted to infants and young children. 

## 14. Overview and Next Steps

The expanding corporate interests in infant and young child nutritional supplements [15,56,57,58] has occurred in the relative absence of oversight. This is not without human consequences. In spite of concerns over infant formula advertising and neonatal marketing within hospitals, 70% of U.S. maternity units participated in formula company marketing programs, and one in four hospitals provided formula supplements to more than half of all healthy, full-term infants prior to discharge in 2007 [110]. As exposure to these products rises, this may not be a small indiscretion. Long-term effects on metabolism and health are sequelae of formula and supplement-induced changes in the infant microbiome [111,112]. At older ages, marketing that suggests busy parents may appreciate that liquid supplements can be used as a child’s “quick breakfast” as “a great way to help them grow” [113] directly takes the items out of their protected status as supplements and into usage as food. The health consequences of such practices should be more seriously considered. 

In the interest of advancing oversight of these products and their consumption patterns a series of action steps will be beneficial: (1) Scientists need to continue their efforts to refine methods permitting the identification of sources and consequences of childhood overweight [114]; (2) clinicians and other healthcare professionals need to play a more substantial role in helping parents assess the validity of product health claims; this may involve educating pediatricians on the science behind the products and their biological effects [115]; and (3) public health and policy professionals need to continue integrating early development of overweight into the larger context of health [116] and consider this specific dietary area and behavior pattern as part of the long-term obesity prevention and reduction agenda [117].

With the United States’ prevalence of obesity among two- to 19-year-olds at 17% [118], and the prevalence of underweight less than 4% [119], it is time to reconsider current oversight of products initially targeted as supplements for small size or clinical repletion among infants and children. Moreover, in a time of expanding global obesity and diabetes, policies governing the health claims of liquid-based nutritional supplements for children with no medically documented signs of illness are important and necessary: the efficacy of wholesale use of supplements is unsubstantiated for the beginning of life, while the long-term impact on health from rapid infant growth or overgrowth is clear. 

As the work of David Barker and numerous scientists have demonstrated, a legacy of generations of undernutrition is relative smallness at birth [1], and the use of calorically-dense supplements to “help” such children grow in infancy may be a distinctly unhealthy effort [120,121]. In fact, rapid infant growth among the relatively small predicts rising chronic disease risks among adults [1,3,4,5,9,25].

In summary, the infant nutritional supplement industry weaves a tapestry of hyperbole promising enhanced growth and development from their products, with ubiquitous claims of promoting “healthy” growth. Substantial evidence identifies that this is scientifically questionable, if not indefensible. While the competitive supplement products try to out-do one another with proprietary claims, the nomenclature “supplements” circumvents much of the FDA oversight. The reality is that the products are being consumed as food and the “supplementary” feedings are associated with weight gain, which is neither growth nor healthy. The evidence at hand identifies potential harm and no one is protecting the children.

## 15. Conclusions

The infant liquid supplement industry is rapidly expanding globally at the expense of vulnerable populations. Aiding and promoting the next generation’s health includes calling on scientists, clinicians, and health care agencies globally [122] to work together and step up to the policy challenges that impact individuals from the youngest ages. It is time for the medical and scientific community to recognize the attention needed here on two levels. First, an examination of the infant supplement industry’s credibility is due, with greater rigor demanded in both the scientific aspects of studies designed to assess growth effects [123] and oversight at multiple levels of product development and use. Second, in the face of the burgeoning evidence documenting the developmental origins of health for a lifetime in patterns of early growth, the supplement industry needs to be held accountable for its impact on each new generation. Augmenting rapid growth among infants and young children is not merely a risk to the global burden of obesity. Among populations who have suffered generations of undernutrition, augmenting infant and young child growth rates is an additional risk for the emergence of chronic disease and reduced health in later life. 
